# Review of Online Evidence-based Practice Point-of-Care Information Summary Providers: Authors’ Reply to the Response by the Publisher of DynaMed

**DOI:** 10.2196/jmir.1626

**Published:** 2010-09-09

**Authors:** Rita Banzi, Alessandro Liberati, Ivan Moschetti, Ludovica Tagliabue, Lorenzo Moja

**Affiliations:** ^3^University of MilanMilanItaly; ^2^University of Modena and Reggio EmiliaModenaItaly; ^1^Italian Cochrane CentreMario Negri Institute for Pharmacological ResearchMilanItaly

## Abstract

We are pleased that the publisher of DynaMed clarified his evidence-based methodology in response to our review. We stress again how good reporting is a prerequisite for transparency. This lesson comes from the reporting of research findings but its extension to the development of information sources should be considered.

We thank Dr. Alper for his comment in response to our review [1], which gives us the opportunity to stress again how essential good reporting is for transparency. This lesson comes from the reporting of research findings, but its extension to the development of information sources should be considered.

Methodologic quality is closely intertwined with the quality of reporting [2]. Lack of details on how research (or in this case: editorial processes) is conducted leads users to assume that the quality was inadequate, unless information to the contrary is provided (the “guilty until proven innocent” approach) [3]. This is often justified because faulty reporting generally reflects faulty methods [4, 5].

We appreciate that the editor of DynaMed (which is a “blockbuster” in the point-of-care information service market) shows the willingness to improve the explicitness and transparency of their methodology. A clear reference on the freely accessible website pages helps users and purchasers to better understand the value of the product.

It is reassuring when well-known and leading publishers do not take for granted their value. Reputation itself is no guarantee for quality. We hope that other publishers will be equally transparent and responsive to criticism.
